# Epigenetic Regulation of Autophagy in Breast Cancer: Implications for Biomarker Discovery and Personalized Therapy

**DOI:** 10.1002/cnr2.70389

**Published:** 2025-12-02

**Authors:** Bushra Faryal

**Affiliations:** ^1^ Department of Precision Medicine University of Campania “Luigi Vanvitelli,” Naples Italy

**Keywords:** autophagy, biomarkers, breast cancer, drug discovery, epigenetics, HDACs, therapy resistance

## Abstract

**Background:**

Breast cancer remains one of the most common and lethal malignancies among women. Despite advanced targeted therapies and precision medicine, therapeutic resistance continues to undermine durable clinical responses. Increasing evidence links epigenetic dysregulation and autophagy as central contributors to breast cancer progression, therapy resistance, and metabolic changes. Histone modifications, non‐coding RNAs, and DNA methylation dynamically regulate autophagy‐related genes (*ATGs*), while autophagy itself co‐regulates the epigenetic landscape under chemotherapeutic stress. This two‐way interplay determines tumor cell fate, influencing sensitivity to chemotherapy, endocrine therapy, and targeted agents.

**Aims:**

This article reviews recent studies on epigenetic mechanisms modulating autophagy and their impact on resistance pathways in breast cancer. Furthermore, this article highlighted the emerging role of epigenetic‐autophagy as a biomarker for early detection, disease monitoring, and predicting therapeutic response.

**Conclusion:**

Finally, the review outlined new therapeutic methods that combine epigenetic modulators and autophagy inhibitors with particular attention to AI‐driven drug discovery and precision oncology. Collectively, this review emphasizes the potential of targeting epigenetic–autophagy crosstalk to overcome therapy resistance and improve patient outcomes.

## 1 Introduction

Breast cancer is the most frequently diagnosed malignancy in women worldwide, with approximately 2.3 million new cases reported in 2022 alone [1]. It is now surpassing lung cancer as the leading cause of cancer‐related mortality among women, ranking 4th globally across all cancer types [2].

In the United States, 2023 statistics estimated 300 590 new cases and 43 700 deaths from invasive breast cancer, accounting for approximately 30% of all cancers in women [3]. Breast cancer is heterogeneous, expressing distinct molecular subtypes that influence tumorigenic potential, progression of the disease, and therapeutic response [4]. Despite advances in precision medicine, therapy resistance remains a challenge, driving tumor recurrence, metastasis, and poor survival outcomes [5].

Epigenetic reprogramming and autophagy have emerged as key regulators of breast cancer biology [6]. Epigenetic mechanisms such as DNA methylation, histone modifications, and non‐coding RNAs control chromatin accessibility and transcriptional regulation without altering the DNA sequence [7, 8]. Aberrant epigenetic changes, such as hypermethylation of tumor suppressor promoters or oncogenic histone modifications, not only drive tumor initiation but also confer adaptive changes under therapy‐induced stress (chemotherapy, radiotherapy, endocrine, and targeted therapies) [9]. The reversibility of these epigenetic alterations highlights their relevance, as targeted interventions can potentially restore treatment sensitivity [10].

Autophagy, an evolutionarily conserved catabolic process, maintains cellular homeostasis by degrading damaged organelles and misfolded proteins [11]. It plays a dual role in cancer, acting as a tumor suppressor in early stages without changing genomic integrity, yet promoting tumors in later stages by supporting survival under hypoxia, starvation, and chemotherapy, which induces selective pressure [12]. This dual functionality complicates therapeutic strategies but also provides opportunities for cancer stage‐specific interventions [13].

The dual role of autophagy across cancer stages and corresponding epigenetic modulations is summarized in Table 1.

**TABLE 1:** Dual role of autophagy in breast cancer.

Cancer stage	Autophagy role	Epigenetic modulation	Impact on therapy response	References
Early	Tumor‐suppressive—removes damaged organelles, reduces genomic instability	Hypermethylation of oncogenes, acetylation of tumor suppressor ATGs (e.g., BECN1 active)	↑ Sensitivity to therapy; improved prognosis	[14, 15]
Late	Tumor‐promoting — metabolic adaptation, stress resistance	Hypomethylation of ATGs, HDAC‐mediated repression of apoptosis, and ncRNAs promoting survival autophagy	↑ Resistance to chemotherapy, endocrine therapy, and targeted agents	[16]

Growing evidence suggests that epigenetic regulation and autophagy are not isolated processes but are closely linked [17]. Epigenetic modifications directly influence the ATGs (autophagy‐related genes) expression, while autophagy modulates chromatin dynamics and post‐translational modifications under cellular and metabolic stress [18]. This two‐way relationship shapes tumor progression and chemotherapy response, defining its importance as both a mechanistic driver and a therapeutic target in breast cancer.

**FIGURE 1:**
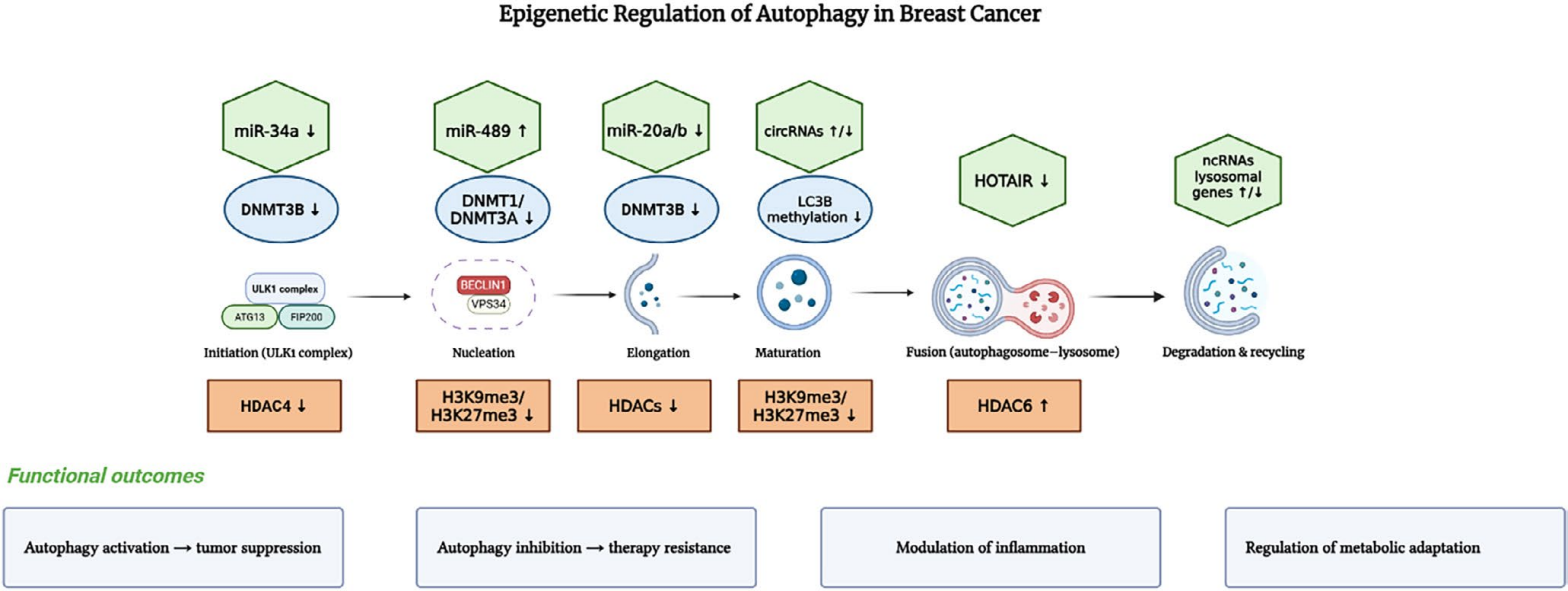
Autophagy pathway and its regulation by epigenetic mechanisms in breast cancer. The process progresses from initiation (ULK1 complex activation) to nucleation (Beclin‐1/VPS34 complex), elongation (ATG5–ATG12–ATG16L1 complex), maturation (LC3 lipidation), fusion with the lysosome, and final degradation/recycling. Epigenetic regulators are indicated: DNA methylation by DNMTs (blue ovals), histone modifications (orange rectangles), and non‐coding RNAs (green hexagons). Upward arrows (↑) denote activation, downward arrows (↓) denote suppression of autophagy. Examples include DNMT3B‐mediated Beclin1 hypermethylation, HDAC4‐mediated ULK1 repression, and miR‐34a targeting of ATG4B. Functional outcomes range from tumor suppression to therapy resistance, inflammation modulation, and metabolic adaptation.

This review focuses on how epigenetic regulation of autophagy contributes to therapy resistance in breast cancer, with a focus on histone modification, DNA methylation, and non‐coding RNAs. Figure 1 provides a schematic overview of the autophagy pathway, highlighting representative epigenetic regulators at different stages of the autophagic pathway in breast cancer.

The review also outlined emerging biomarkers derived from these pathways and addressed therapeutic approaches that combine epigenetic and autophagy modulation.

## 2 Epigenetic Regulation of Autophagy in Breast Cancer

Epigenetic alterations such as DNA methylation, histone modifications, and non‐coding RNAs significantly influence autophagy‐related genes *(ATGs)* and thereby shape tumor behavior, therapy response, and disease progression [19]. These mechanisms either silence tumor‐suppressive autophagy in early tumorigenesis or, oppositely, enhance pro‐survival autophagy under therapeutic stress. Table 2 provides a comparative perspective on stage‐specific effects [23].

**TABLE 2:** Cancer stage‐dependent epigenetic effects on autophagy.

Epigenetic mechanism	Early‐stage effect	Late‐stage effect	Example genes/regulators	References
DNA methylation	Hypermethylation of oncogenes; hypomethylation of tumor suppressors	Hypomethylation of ATGs promotes survival	*BECN1, LC3B, ATG5*	[20]
Histone modifications	Acetylation of pro‐autophagy genes → activation	HDAC‐mediated deacetylation → suppression of apoptosis	*H3K9ac, H3K27me3*	[21]
Non‐coding RNAs	Tumor‐suppressive miRNAs activate autophagy	Oncogenic lncRNAs/circRNAs inhibit apoptosis, promote survival autophagy	*miR‐34a, HOTAIR, circCDYL*	[22]

### 2.1 DNA Methylation and Autophagy Genes

DNA methylation primarily occurs at CpG islands within promoter regions and is a central epigenetic mechanism regulating *ATG* transcription [24]. In the context of autophagy, aberrant methylation of autophagy‐related genes *(ATGs)* can disrupt key steps in the autophagic process, from initiation to autophagosome maturation [25]. Hypermethylation generally silences tumor suppressive *ATGs*, while hypomethylation may activate survival‐promoting autophagy, especially in resistant cancer [26].

In breast cancer, hypermethylation of ***BECN1***, ***LC3B***, and ***ATG5*** is frequently observed in early‐stage tumors, resulting in reduced autophagic activity, accumulation of damaged organelles, genomic instability, and tumor initiation [27]. In contrast, hypomethylation of these same genes is often seen in advanced diseases, particularly in triple‐negative breast cancer (***TNBC***), where it enhances autophagic flux and supports metabolic adaptation and resistance to chemotherapy‐induced oxidative stress and hypoxia [28]. Similar patterns are observed for ***RASSF1A*** and ***DAPK1***, whose promoter methylation correlates with aggressive phenotypes and a poor prognosis [29, 30].

The reversible nature of DNA methylation makes it an attractive target for therapeutic intervention [31]. Demethylating agents such as ***azacitidine*** and ***decitabine*** can restore the ATG expression in early‐stage disease, potentially reactivating the tumor suppressor function of autophagy [32, 33]. In advanced breast cancer, where autophagy promotes cell survival, the clinical rationale favors combining epigenetic modulators with autophagy inhibitors (e.g., ***chloroquine*** and ***hydroxychloroquine***) [34, 35]. For example, in advanced triple‐negative breast cancer, reactivation of autophagy under DNA damage and chemotherapy induces stress, acting as a protective mechanism by supplying metabolic substrates, mitigating cellular damage, and preventing apoptosis, thereby promoting therapy resistance [36]. This stage‐specific strategy underscores the need for precise patient classification based on methylation profiling [37].

### 2.2 Histone Modifications and Autophagy Regulation

Histone modifications, including acetylation, methylation, phosphorylation, and ubiquitination, regulate chromatin structure and transcriptional accessibility of *ATGs* [38]. Histone acetyl transferases (***HATs***) and histone deacetylases (***HDACs***) regulate lysine acetylation, with acetylation generally promoting transcription and *HDAC*‐mediated deacetylation repressing it [39]. Similarly, histone methylation can either activate or repress gene transcription depending on the specific residues and methylation patterns, such as ***H3K4me3*** as an activating mark vs. ***H3K27me3*** as a repressive one [40].

In breast cancer, dysregulated histone modification (**acetylation**/**methylation**) disrupts autophagy regulation. For example, higher ***HDAC*** activity silences *ATGs* such as ***BECN1*** and ***LC3B***, suppressing autophagic flux [41]. In contrast, hyperacetylation of certain histone marks can activate autophagy genes, which in later disease stages may promote survival under chemotherapy‐induced oxidative and DNA damage stress, a phenomenon observed particularly in TNBC. Moreover, histone methyltransferases (***HMTs***) like ***EZH2*** deposit repressive ***H3K27me3*** marks on *ATG* promoters, contributing to therapy resistance, whereas demethylases such as ***KDM6B*** remove these marks and restore autophagy [42].

Given their druggability, histone‐modifying enzymes are of higher therapeutic interest. Histone deacetylase inhibitors (***HDACi***), including ***vorinostat***/***SAHA*** (pan‐HDAC inhibitor), ***LBH589***/***Panobinostat***, and ***EZH2*** (Enhancer of zeste homolog 2) inhibitors, for example, ***tazemetostat***, can restore acetylation and reactivate silenced *ATGs*, restoring autophagy's tumor‐suppressive role in early stages of cancer [43]. However, in later stages or chemoresistant TNBC, these same agents may accidentally enhance the pro‐survival role of autophagy. In such cases, combining ***HDAC*** inhibitors or ***HMT*** inhibitors targeting ***EZH2*** with autophagy inhibitors like **chloroquine** (CQ) could block the protective mechanism and resensitize tumors to chemotherapy. This approach highlights the critical importance of histone modification profiling to guide therapeutic selection [44].

### 2.3 Role of Non‐Coding RNAs in Autophagy Modulation

Non‐coding RNAs (***ncRNAs***), including microRNAs (***miRNAs***), long non‐coding RNAs (***lncRNAs***), and circular RNAs (***circRNAs***), primarily regulate autophagy at the post‐transcriptional level [45]. By modulating the expression of *ATGs* and associated upstream signaling pathways, *ncRNAs* influence different stages of the autophagic process (initiation, autophagosome formation, and maturation). For instance, several *ncRNAs* suppress ***mTOR*** signaling, thereby activating or promoting the phosphorylation of upstream initiators such as ***ULK1***, triggering autophagy initiation [7]. Depending on their specific targets, ncRNAs can either encourage or inhibit autophagic activities, thus affecting cancer development and resistance to therapy [46].

In breast cancer, several ***miRNAs*** negatively regulate autophagy, thereby suppressing autophagic flux and impairing chemotherapy sensitivity. Some examples are ***miR‐96‐5p***, ***miR‐20a/20b***, ***miR‐34a***, ***miR‐25***, ***miR‐129‐5p***, and ***miR‐200c***, which suppress autophagy by downregulating key ATG regulators, and these inhibitory ***miRNAs*** are summarized in Table 3 [52].

**TABLE 3:** miRNAs associated with autophagy and their mechanism of action.

miRNA	Mechanism	Impact on therapy	References
miR‐20a/20b	Downregulates RB1CC1/FIP200	Reduced rapamycin‐induced autophagy	[47]
miR‐96‐5p	‐ Inhibits FOXO1‐mediated autophagy—Inhibits autophagic flux by blocking LC3 conversion and p62 degradation in MCF‐7, MDA‐MB231	Reduces apoptosis resistance and enhances cancer cell proliferation	[48]
miR‐34a	Downregulates key autophagosome proteins, Beclin1, LC3I/II, and ATG4B.	Enhances the chemoresistance of doxorubicin and topotecan drugs.	[49]
miR‐25	Suppresses ULK1‐dependent autophagy	Decreases in ISL‐induced chemosensitivity in breast cancer cells	[47]
miR‐129‐5p	Targets HMGB1 to block autophagy	Sensitize cells to radiotherapy	[50]
miR‐200c	Targets UBQLN1 to block autophagy	Resensitize cells to radio resistance	[51]

In contrast, certain ***miRNAs***, such as ***miR‐489***, promote autophagy by targeting ***LAPTM4B*** (lysosome‐associated transmembrane protein 4B), which can reverse drug resistance in breast cancer [53]. The functional consequences of ***miRNA*** expression are context‐dependent in some cases, as higher autophagic flux supports tumor survival, but, oppositely, it induces autophagic cell death and improves therapy outcomes [54].

lncRNAs also play an important role in autophagy regulation [55]. The oncogenic ***lncRNA HOTAIR*** inhibits autophagy in breast cancer via chromatin remodeling and activation of the ***PI3K/AKT/mTOR*** pathway [56, 57]. This repression involves ***mTOR***‐mediated phosphorylation and inhibition of autophagy, initiating kinases such as ***ULK1*** [58]. ***HOTAIR*** also acts as a competing endogenous RNA (***ceRNA***), sequestering ***miR‐34a***, a tumor suppressor that targets ***ATG4B***, ***Beclin‐1***, and ***HMGB*** (high mobility group box) proteins, which is an anti‐autophagic ***miRNA*** in breast cancer [56]. ***HOTAIR's*** main effect is to suppress autophagy via ***mTOR*** activation, a mechanism that contributes to metastasis and chemotherapy resistance [59]. Silencing ***HOTAIR*** can restore autophagy and increase chemotherapy sensitivity. Knockdown of lncRNA HOTAIR downregulates the drug resistance of breast cancer cells to doxorubicin via the PI3K/AKT/mTOR signaling pathway [60].

Circular RNAs (***circRNAs***) have recently emerged as additional regulators of autophagy. By sponging ***miRNAs***, ***circRNAs*** modulate both pro and anti‐autophagic pathways at transcriptional, post‐transcriptional, and epigenetic levels [61, 62]. Some examples of ***circRNAs*** involved in breast cancer autophagy regulation are listed in Table 4.

**TABLE 4:** Examples of circRNA and their mechanisms of action are described.

circRNA	Disease context	Mechanism	Outcome	References
circHECTD1	Ischemic Stroke	Sponges miR‐142 to upregulate *TIPARP*, activating astrocyte autophagy	Aggravates neuronal injury	[63]
circCDYL	Breast Cancer	Binds miR‐1275, elevating ATG7/ULK1 and promoting autophagy	Enhances tumor growth and metastasis	[61]
circ‐Dnmt1	Breast Cancer	Interference with p53/AUF1 nuclear localization to induce autophagy	Promotes cancer cell proliferation	[64]

The reversibility of ***ncRNA***‐mediated regulation offers several opportunities for therapeutic interventions, including restoring tumor suppressive ***miRNAs*** (e.g., ***miR‐489***) could promote autophagic cell death in chemoresistant tumors, by inhibiting oncogenic ***miRNAs***, for example, anti‐miRs targeting ***miR‐200 or miR‐96‐5p***, or deploying antisense oligonucleotides against oncogenic lncRNA such as HOTAIR [65].

Similarly, manipulating ***circRNA‐miRNA*** networks may selectively inhibit pro‐survival autophagy or increase cytotoxic autophagy, depending on the disease/cancer stage and therapeutic context [66].

## 3 Epigenetic and Autophagy Biomarkers in Breast Cancer: Clinical Implications

Epigenetic alterations and autophagy‐related changes not only affect breast cancer biology but also have potential as clinically relevant biomarkers. These molecular signatures can aid in early detection, predict therapeutic responses, and guide treatment choices [67]. While their mechanistic basis was covered in Section 2, this discussion emphasizes their translational and prognostic importance.

### 3.1 Methylation Signatures of Autophagy‐Related Genes in Early Breast Cancer Detection

DNA methylation patterns of ATGs represent promising biomarkers for breast cancer diagnosis and progression [68].

In early‐stage breast cancer, hypermethylation of ***BECN1***, ***LC3B***, and ***ATG5*** suppresses autophagic activity, contributing to tumorigenesis. In advanced breast cancer, particularly ***TNBC***, hypomethylation of these genes enhances autophagic flux and chemotherapy resistance [69]. This stage‐dependent switch reflects a broader dual role of autophagy in breast cancer, with implications for early therapy selection and timing [70].

Likewise, promoter methylation of ***RASSF1A*** and ***DAPK1*** is associated with aggressive phenotypes and poor prognosis, especially in ***ER*** + tumors. Hypomethylation patterns in invasive breast cancer often activate ***EMT***‐associated pathways (e.g., ***SMAD3***, ***STAT3***, ***PLAU***, and ***VIM***) [71].

These methylation signatures can be detected in circulating tumor DNA (ctDNA), particularly in liquid biopsies, making them a non‐invasive biomarker for early detection and breast cancer subtype stratification [72]. Longitudinal profiling of methylation states may provide real‐time insight into evolving therapy resistance, such as chemotherapy (taxanes and anthracyclins), endocrine therapy (tamoxifen), radiotherapy, and targeted therapies (*PARP* inhibitors and *HER2* inhibitors). where autophagy acts as a survival mechanism under treatment‐induced selective stress [73].

### 3.2 Histone Modifications as Prognostic Biomarkers

Histone modifications, including acetylation and methylation, act as prognostic markers in breast cancer by regulating chromatin accessibility and transcriptional activity. Specific histone modification patterns correlate strongly with disease subtype, progression, and therapeutic outcomes [74].

For example, elevated tri‐methylk27 (**H3K27me3**) marks at the promoter region of autophagic genes (***ATG5*** and ***BECN1***) are associated with ***TNBC*** subtypes such as ***MDA‐MB‐231*** and are linked to poor prognosis [75]. Similarly, elevated levels of trimethyl‐H3K9 (**H3K9me3**) promote gene silencing, whereas acetylation of H3K9 (**H3K9ac**) supports active transcription [69]. An imbalance between these opposite modifications alters autophagy and increases mechanistic potential.

By contrast, hyper‐acetylated **H3K18ac**, **H4K12ac**, along with hypermethylated **H3K4me2** and **H4K20me3**, are characteristics of luminal‐like subtypes such as ***MCF7***, and are associated with favorable outcomes [76]. Loss of acetylated H4K16 **(H4K16ac)** has emerged as an early sign of breast cancer development in most cases and is strongly associated with genomic instability and tumor aggressiveness [77].

Chromatin‐immunoprecipitation (**ChIP**) analyses further confirmed subtype‐specific alterations: breast cancer tissues showed increased **H3K9me3** at centromeric satellites (***SAT2***), and **H4K20me** at pericentric heterochromatin, both biomarkers of transcriptional repression, and chromatin compaction [78].

Among histone‐modifying enzymes, ***HDAC6*** (histone deacetylase 6) represents a particularly notable biomarker. ***HDAC6*** is frequently overexpressed in ER+ breast cancers (such as ***MCF‐7***, ***ZR‐75‐1***, and ***T‐47D***), where it correlates with low tumor grade, smaller size, and hormone receptor positivity [79, 80]. Clinically, higher expression of ***HDAC6*** is linked to improved disease‐free survival and increased responsiveness to tamoxifen, making it a potential prognostic and predictive biomarker in endocrine‐responsive cancers. Interestingly, ***HDAC6*** represses *ATGs* such as ***BECN1*** and ***LC3B*** and is often detected in resistant tumors; its expression paradoxically aligns with favorable clinical outcomes in **ER**+ disease, suggesting a context‐dependent role [81].

Altogether, these findings highlight that profiling histone modifications in tumor tissues or circulating nucleosomes holds considerable promise for predicting cancer progression, anticipating therapeutic resistance, and tailoring personalized therapeutic strategies in breast cancer [82].

Table 5 summarizes exemplary epigenetic mechanisms that regulate autophagy, their target genes or proteins, functional impact on autophagy, associated roles in drug resistance, and therapeutic strategies under preclinical or clinical trials.

**TABLE 5:** Representative epigenetic mechanisms regulating autophagy in breast cancer and their therapeutic implications.

Epigenetic mechanism	Target genes/proteins	Functional impact on autophagy	Role in drug resistance	Therapeutic strategy	Example/outcome	References
H3K27me3 (via EZH2)	*ATG5, BECN1*	Silences autophagy genes	Limits autophagic flux, promotes resistance	EZH2 inhibitors (e.g., tazemetostat) + autophagy inhibitors (CQ/HCQ)	Restores ATG5 expression and enhances chemosensitivity in TNBC and glioblastoma models	[42]
HDAC‐mediated deacetylation	*ATG7, LC3B*	Suppresses autophagy gene expression	Reduces autophagosome formation, increases survival	HDAC inhibitors (e.g., SAHA) + chemotherapy	SAHA + doxorubicin elevates LC3B‐II and triggers autophagic cell death in TNBC	[83]
DNA Hypermethylation	*BECN1, ATG5, LC3B*	Silences promoters, inhibits autophagy	Promotes survival of chemotherapy‐resistant cells	DNMT inhibitors (e.g., 5‐azacytidine) + autophagy inhibitors	5‐azacytidine reactivates *BECN1* and synergizes with chloroquine (shown in leukemia models; concept under study in breast cancer)	[84]
Combination therapy	Autophagy machinery	Blocks cytoprotective autophagy	Restores chemosensitivity	Autophagy inhibitors (CQ/HCQ) + HDAC/DNMT inhibitors	HCQ + SAHA reduces tumor growth in breast cancer PDX models	[35]

### 3.3 ncRNAs As Predictive Biomarkers

Non‐coding RNAs have emerged as versatile predictive markers. Among ***miRNAs***, ***miR‐34a***, ***miR‐489***, and ***miR‐200c*** are particularly important. Upregulated ***miR‐34a*** and ***miR‐200c*** levels correlate with reduced autophagy and poor chemotherapy response, whereas higher ***miR‐489*** expression enhances autophagy and restores chemosensitivity [85].

Similarly, the ***lncRNA HOTAIR*** has strong prognostic and predictive value: its overexpression predicts resistance to endocrine therapy and increased metastatic potential [86]. ***CircRNAs***, though less studied, are gaining attention as stable molecules in circulation that can modulate autophagy indirectly through **miRNA** sponging [87].

Incorporating panels of ***ncRNAs*** into diagnostic workflows may refine patient stratification and enable personalized therapy selection based on predicted autophagy activity [88].

### 3.4 Integrated Biomarker Potential

**FIGURE 2:**
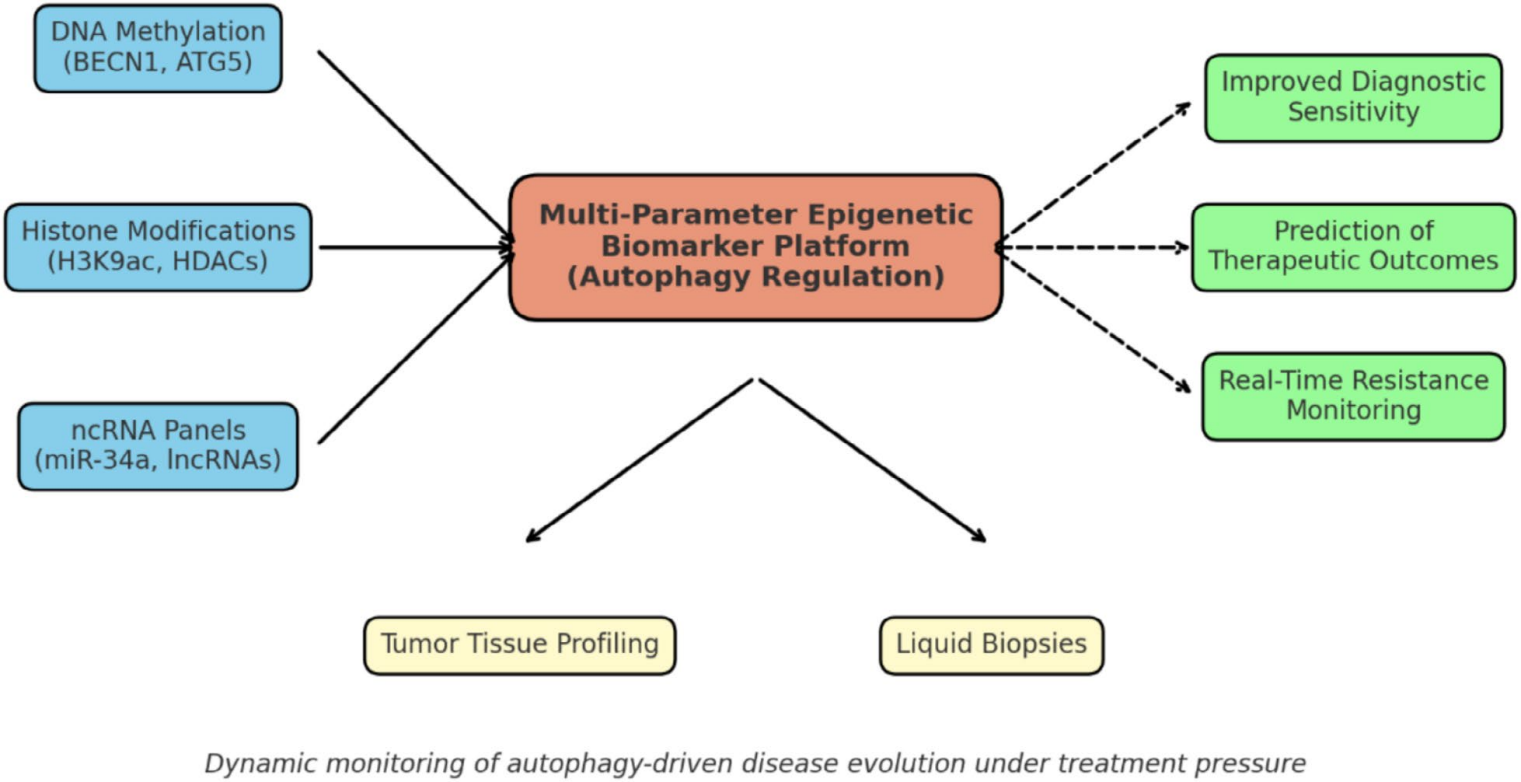
Epigenetic regulation of autophagy as a biomarker platform in breast cancer. The integration of DNA methylation, histone modifications, and ncRNA panels into a multi‐parameter platform enhances diagnostic sensitivity, predicts therapeutic outcomes, and enables real‐time resistance monitoring from both tumor tissues and liquid biopsies.

The highest potential lies in combining methylation profiles, histone modification signatures, and ***ncRNA*** panels into multi‐parameter biomarker platforms. Such integrative approaches could improve diagnostic sensitivity, predict therapeutic outcomes, and track resistance in real time [89]. Importantly, profiling these biomarkers in tumor tissue and liquid biopsies may facilitate dynamic monitoring of disease evolution under treatment pressure Figure 2.

## 4 Role of Autophagy in Therapy Resistance

Autophagy is a key adaptive mechanism that enables breast cancer cells to tolerate chemotherapy‐induced selective pressure by modulating cellular stress responses, apoptosis evasion, and metabolic adaptation [90]. Its role in breast cancer is context‐dependent: while early autophagy suppression contributes to tumor initiation, once established, breast cancers frequently exploit autophagy as a pro‐survival mechanism during chemotherapy, radiotherapy, endocrine, and targeted therapy. Thus, autophagy modulation represents both a barrier to effective treatment efficacy and a therapeutic opportunity [91].

**FIGURE 3:**
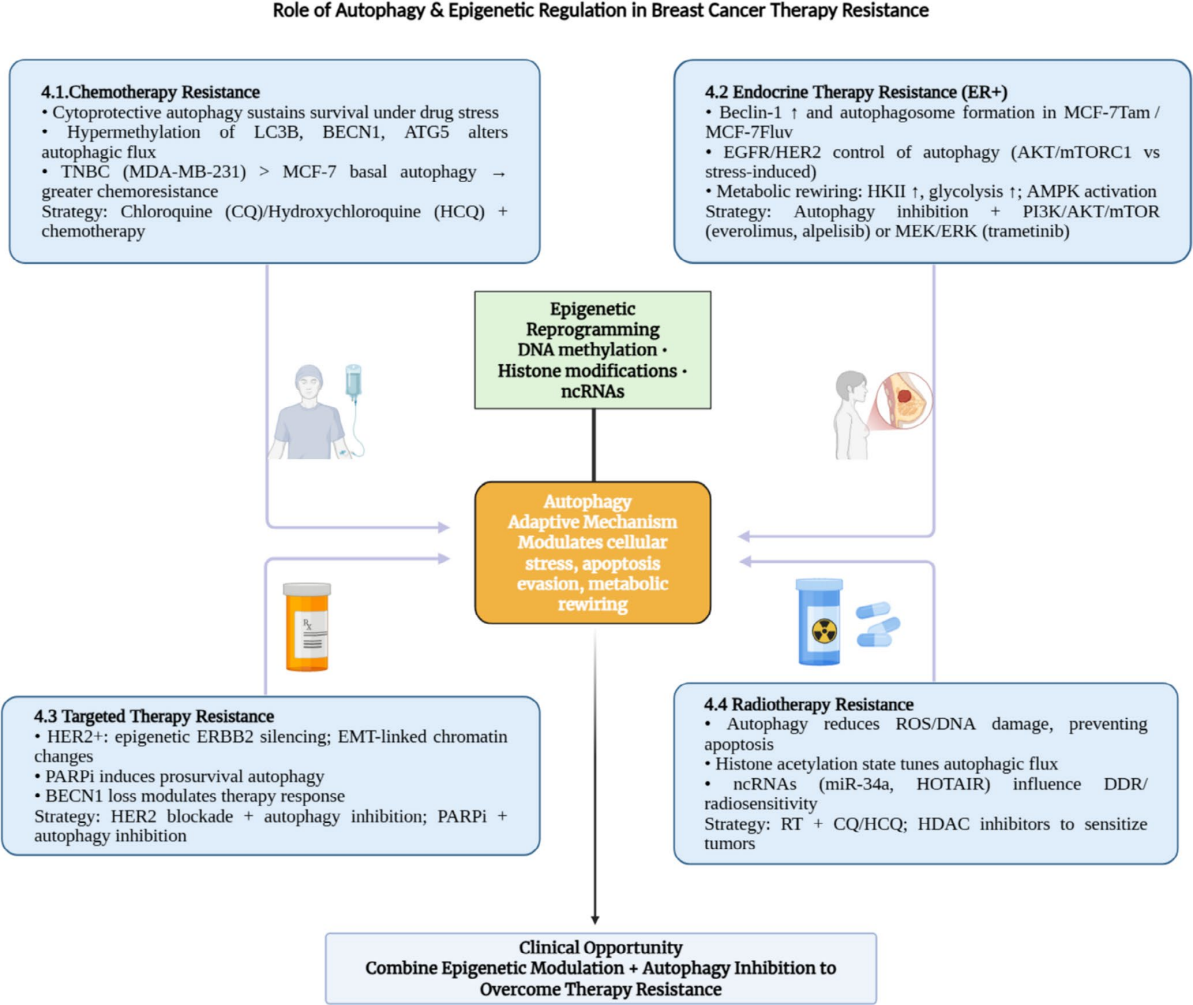
Schematic overview of autophagy and epigenetic regulation in breast cancer therapy resistance. Autophagy, while tumor‐suppressive in early stages, promotes survival under chemotherapy, endocrine therapy, targeted therapy, and radiotherapy. Epigenetic alterations (DNA methylation, histone modifications, non‐coding RNAs) reshape autophagy‐related gene expression (e.g., BECN1, ATG5, and LC3B), driving apoptosis evasion and metabolic adaptation. Therapeutic strategies such as autophagy inhibitors (chloroquine, hydroxychloroquine) and combined pathway blockade (PI3K/mTOR, MEK/ERK, and EGFR/HER2) show promise in resensitizing resistant breast cancer cells.

The following Subsections 4.1, 4.4 (Figure 3) summarize how autophagy and epigenetic regulation collectively cause therapy resistance in breast cancer, along with therapeutic strategies to overcome this barrier through combined targeted treatments.

### 4.1 Chemotherapy Resistance

Chemotherapy is a first‐line treatment of several breast cancer subtypes, yet the emergence of chemoresistance poses a major clinical challenge. Resistance arises from diverse mechanisms, drug target alterations, drug inactivation, epithelial to mesenchymal transition (***EMT***), cancer stem cell enrichment, and evasion of apoptosis [92].

In this context, autophagy frequently functions as a cytoprotective mechanism allowing breast cancer cells to survive chemotherapy‐induced stress. For example, ***TNBC*** cell lines such as ***MDA‐MB‐231*** show higher basal autophagy under stress than luminal ***MCF‐7*** cells, correlating with higher chemoresistance [93]. Epigenetic reprogramming can enhance this pro‐survival autophagy, enabling tumor cells to overcome drug‐induced cytotoxicity [94]. Epigenetic alterations contribute directly to chemotherapy resistance by reshaping autophagic flux [95].

Late‐stage tumors, particularly ***TNBC***, often show hypermethylation of key autophagic genes such *as **LC3B***, ***BECN1***, and ***ATG5***, which disrupt autophagic flux and promote chemoresistance. Experimental knockdown of ***ATG5**/**7*** or pharmacological inhibition of autophagy with chloroquine (CQ) has been shown to resensitize resistant breast cancer cells to chemotherapy [96].

Collectively, these findings support the therapeutic potential of combining autophagy inhibitors with standard chemotherapy to overcome epigenetically driven drug resistance, especially in aggressive cancer subtypes with poor prognosis [97].

### 4.2 Endocrine Therapy Resistance

Endocrine therapy, including selective estrogen receptor modulators (***SERMs***) like tamoxifen and selective estrogen receptor degraders (***SERDs***) such as fulvestrant, is standard treatment for treating estrogen receptor‐positive (***ER*** +) breast cancer [98]. However, resistance remains a significant clinical challenge, contributing to poor prognosis and relapse.

Autophagy has emerged as a critical mediator of endocrine resistance by enabling cancer cells to survive therapeutic stress, avoid apoptosis, and maintain metabolic plasticity [99, 100].

Endocrine‐resistant derivatives of ***MCF**‐**7*** cells, such as ***MCF‐7Tam*** (tamoxifen resistance) and ***MCF‐7Fluv*** (fulvestrant resistance), showed higher autophagy, characterized by increased ***Beclin‐1*** expression and increased autophagosome formation. These changes are closely related to activation of ***EGFR*** and ***HER2*** signaling, which directly interact with autophagy‐related proteins such as ***Beclin‐1*** [101].

These signaling pathways (***EGFR/HER2***) have a context‐dependent regulation of autophagy. Under nutrient‐rich conditions, ***EGFR*** suppresses autophagy through the ***AKT/mTORC1*** pathway, whereas under metabolic stress, ***EGFR*** promotes autophagy by releasing Rubicon from ***Beclin‐1*** [102]. Such plasticity allows resistant ***ER*** + cells to survive both metabolic deprivation and endocrine therapy [103].

Certain metabolic pathways also promote resistance, such as higher hexokinase II expression and glycolysis rates in tamoxifen‐resistant cells, reduced mTOR‐S6K signaling, and activation of autophagy. Additionally, metastasis‐associated proteins trigger AMPK, further promoting pro‐survival autophagy [104].

These mechanistic insights point toward therapeutic opportunities, that is, dual inhibition of signaling pathways and autophagy is a promising approach:

- Lapatinib, a dual EGFR/HER2 inhibitor, has shown potential but is limited by compensating autophagic processes [105].
- PI3K/AKT/mTOR (everolimus, alpelisib) and MEK/ERK inhibitors (trametinib) suppress oncogenic signaling and autophagic survival with synergistic effects when combined [106, 107].
- However, toxicity and secondary resistance remain key barriers, highlighting the need for patient‐tailored strategies.

In summary, endocrine therapy resistance in ER+ breast cancer is maintained by a network of autophagy, growth factors, and metabolic rewiring. Rational combination therapies that integrate autophagy inhibition with PI3K/mTOR or MEK/ERK blockade represent promising avenues to overcome resistance and improve clinical outcomes [108].

### 4.3 Resistance to Targeted Therapies

Resistance to targeted therapies in breast cancer arises from complex interactions among epigenetic, genetic, and adaptive signaling mechanisms. Current strategies include ***PARP*** inhibitors (***talazoparib*** and ***olaparib***) and ***HER2***‐targeted therapies (***lapatinib and trastuzumab***). However, both drug classes face resistance mechanisms that are reinforced by epigenetic modifications and autophagy‐mediated cell survival [105].

In ***HER2**+* disease, resistance can occur through epigenetic silencing of the ***ERBB2** gene* expression, leading to reduced ***HER2*** signaling and diminished responsiveness to lapatinib and trastuzumab [109, 110].

Epigenetic rewiring associated with ***EMT*** (epithelial‐mesenchymal transition) contributes to this resistance by altering the ***ERBB2*** promoter, including reduced **H3Kac/H3K4me** and increased **H3K9ac**, resulting in chromatin compaction and translational repression [111].

Autophagy further modulates ***HER2*** therapy responses [112]. Monoallelic deletion of the ***BECN**‐**1 gene***, which encodes ***Beclin‐1*** protein, is common in ***HER2**+* breast cancer. Loss of ***Beclin‐1*** reduces basal autophagy and may exert a tumor‐suppressive effect during early tumorigenesis [113].

Interestingly, tumors with a partial loss of ***BECN‐1*** express increased sensitivity to ***HER2***‐targeted therapy, suggesting that autophagy inhibition in combination with ***HER2*** blockade may enhance therapeutic efficacy [114].

*PARPi* (*PARP* inhibitor) resistance is also modulated by autophagy [115]. In *BRCA* wild‐type and homologous recombination repair (HRR)‐proficient breast cancers, talazoparib (PARPi) has been shown to induce the stabilization of autophagy initiation factors and enhance autophagosome formation, as well as prosurvival autophagy [116]. This process mitigates therapy‐induced damage by recycling impaired organelles and alleviating stress [116].

In contrast, genetic depletion of *BECN1*, *ATG5*, *p62/SQSTM1*, or *LAMP1* or pharmacological inhibition by chloroquine, resensitizes breast cancer cells to PARPi by shifting DNA repair towards error‐prone NHEJ (non‐homologous end joining), leading to mitotic catastrophe and genomic instability [116].

### 4.4 Radiotherapy Resistance

Radiotherapy is an essential treatment type for localized and advanced breast cancer, exerting its cytotoxic effects primarily through the generation of reactive oxygen species (***ROS***), induction of DNA double‐strand breaks, and mitochondrial damage. Despite its effectiveness, radioresistance remains a significant barrier to durable responses in many breast cancer patients [117].

Autophagy plays a central role in this adaptive resistance. By degrading and recycling damaged organelles and proteins, autophagy mitigates radiation‐induced oxidative stress and prevents apoptosis, thereby enhancing tumor cell survival. Breast cancer cell lines with high basal autophagy, such as triple‐negative subtypes, demonstrate greater resistance to radiation compared with luminal counterparts. Inhibition of key autophagy regulators, including ***ATG5*** and ***Beclin‐1***, or pharmacological inhibition with chloroquine (***CQ***) and hydroxychloroquine (***HCQ***), has been shown to resensitize resistant cells to radiotherapy [36].

Epigenetic modifications also contribute to radiation response. For example, histone hypoacetylation at promoters of autophagy‐related genes represses autophagic flux, while hyperacetylation enhances pro‐survival autophagy under radiation‐induced stress [1]. Similarly, non‐coding RNAs, including ***miR‐34a*** and ***HOTAIR***, modulate autophagy and DNA damage repair pathways, directly influencing radiosensitivity. Dysregulation of these epigenetic networks creates a permissive environment for tumor persistence following radiation exposure [118].

Clinically, these findings suggest that combining radiotherapy with autophagy inhibition and epigenetic modulation represents a promising avenue to overcome radioresistance. For instance, the concurrent use of ***CQ*** with radiotherapy enhances tumor regression in preclinical breast cancer models, while histone deacetylase inhibitors (***HDACi***) can synergize with radiation to alter chromatin accessibility and disrupt DNA repair [119].

In summary, radiotherapy resistance in breast cancer is sustained by the interplay of autophagy and epigenetic reprogramming, which collectively reduce treatment efficacy [120]. Therapeutic strategies that incorporate autophagy inhibitors alongside radiotherapy, particularly in aggressive subtypes such as ***TNBC***, need further clinical investigation [121].

## 5 Epigenetic Inhibitors Targeting Autophagy in Breast Cancer

In breast cancer, therapeutic resistance is frequently driven by adaptive mechanisms involving deregulated autophagy and epigenetic reprogramming [122]. Because these processes are interlinked, dual targeting has emerged as a promising area to overcome therapy resistance, particularly in refractory and aggressive subtypes [123]. This section highlighted how autophagy inhibitors and epigenetic drugs are used individually or in combination to improve therapeutic outcomes.

### 5.1 Epigenetic Drugs (HDAC/DNMT Inhibitors) Modulating Autophagy

Epigenetic alteration of ***ATGs*** contributes significantly to therapy resistance in breast cancer. Promoter hypermethylation and histone deacetylation can silence ***ATG5, BECN1***, and *LC3B*, thereby suppressing autophagy and promoting tumor survival [124, 125]. Unlike genetic alterations, these changes are reversible, which makes them attractive targets for treatment.

DNA methyltransferase inhibitors (***DNMTis***) like decitabine and azacitidine can reverse the promoter methylation, restoring the expression of key autophagy and apoptosis regulators. This reactivation sensitizes resistant breast cancer cells to chemotherapy and endocrine therapies [126].

Likewise, histone deacetylase inhibitors (***HDACis***), including vorinostat and Panobinostat, remodel chromatin to transcriptional accessibility, reactivating silenced autophagy effectors and promoting pro‐death autophagic responses [127].

These “**epidrugs**” are being investigated as monotherapies and in combination with other chemotherapy, endocrine therapy, and immune checkpoint inhibitors, with promising preclinical and early clinical results [128]. However, clinical translation faces challenges, including optimizing dosing regimens, reducing toxicity, and identifying biomarkers that predict responses [129].

Notably, clinical trials such as ***NCT02915523*** investigate ***Decitabine*** in combination with ***Chloroquine*** in advanced solid tumors to exploit the synergy between epigenetic reactivation and autophagy inhibition [130].

### 5.2 Autophagy Inhibitors as Sensitizers to Epigenetic Therapy

While autophagy can initially act as a tumor suppressor, in established breast cancers it frequently functions as a survival mechanism under therapeutic stress. Consequently, autophagy inhibition becomes an attractive strategy to enhance the cytotoxic effects of epigenetic therapy [131].

Chloroquine (***CQ***) and Hydroxychloroquine (***HCQ***) are FDA‐approved agents that block lysosomal acidification and autophagosome‐lysosome fusion and have been shown to enhance the efficacy of **DNMTis** and ***HDACis*** [132].

Mechanistically, chloroquine amplifies DNA damage through reactive oxygen species (***ROS***), mediated double‐strand breaks, while histone deacetylase inhibitors impair homologous recombination repair, creating synthetic lethality [133, 134]. Preclinical studies demonstrate that combining ***CQ*** with ***HDACi***, such as ***Panobinostat***, induces synergistic apoptosis and tumor regression in ***TNBC*** models. In this context, ***Panobinostat*** triggers endoplasmic reticulum stress and apoptosis but simultaneously induces protective autophagy, which ***CQ*** effectively blocks [132, 135, 136, 137]. This dual disruption results in the accumulation of misfolded proteins, ***p62***, and polyubiquitylated aggregates, leading to cell death in ***TNBC*** cell lines such as ***MDA‐MB‐231*** and ***SUM159PT*** [138]. Ongoing clinical trials, including ***NCT02378532***, are currently testing these combinations in patients with advanced breast cancer, highlighting the translational potential of this approach [139].

### 5.3 Combination Therapies for Personalized Medicine in Breast Cancer

The heterogeneity of breast cancer involves treatment strategies that integrate epigenetic and autophagy‐related biomarkers for precision medicine. Profiling DNA methylation, histone modification, and non‐coding RNA expression can help in patient classification and predict therapeutic response [126].

Several combined therapies are under investigation Table 6.

**TABLE 6:** Strategies to overcome therapy resistance in breast cancer cells.

Approach	Mechanism	Examples	Outcome	References
Kinase inhibitors	Target overexpressed kinases in resistant clones	Pralsetinib (RET inhibitor)	Suppresses *ESR1* fusion‐driven tumor growth in preclinical models.	[140]
PI3K/mTOR inhibitors	Block reactivated downstream pathways	Everolimus (mTOR inhibitor) + trastuzumab	Improves PFS in HER2+/HR‐ advanced breast cancer.	[141]
Epigenetic modulators	Reverse gene silencing/activation	HDAC inhibitors + DNMT inhibitors	It restores *PTEN* expression and sensitizes cells to therapy.	[142]
Combination therapies	Dual‐pathway inhibition to prevent redundancy	Trastuzumab + PI3K inhibitors (e.g., alpelisib)	Overcomes HER2 L755S resistance; clinical trials are ongoing.	[143]
CSC‐targeted therapies	Induce ferroptosis/pyroptosis or block miR‐27‐3p	Nanoparticles + γ‐secretase inhibitors	Reduces CSC‐driven recurrence in TNBC.	[144]

- ***HDAC*** inhibitors such as ***entinostat***, when combined with immune checkpoint inhibitors like ***anti‐PD1/PD‐L1*** antibodies, have shown increased immunogenic cell death in triple‐negative breast cancer (***TNBC***), leading to better response rates [145].
- Combining ***DNMT*** inhibitors with ***miRNA*** mimics like ***miR‐34a or miR‐200c*** can restore the autophagy regulation and suppress epithelial‐mesenchymal transition, and enhance responsiveness to chemotherapy [146].
- Targeted multi‐drug treatments, such as ***Gedatolisib*** (a ***PI3K/mTOR*** inhibitor) combined with ***Palbociclib*** (***CDK4/6*** inhibitor) and ***Fulvestrant*** (***SERD***), are currently under investigation in the ***VIKTORIA‐1*** trial for metastatic breast cancer [147].
- ***Lasofoxifene*** combined with ***Abemaciclib*** has shown efficacy in treating ***ESR1***‐mutant breast cancers that have become resistant to standard endocrine therapies [148].
- ***Durvalumab + Dato‐DXd*** (an ***ADC***) to combine immunotherapy with targeted cytotoxic in ***TNBC*** [149].
- ***Elacestrant*** combined with ***Alpelisib, Everolimus, or CDK4/6*** inhibitors are being tested in various ongoing trials for advanced ***ER+/HER2*** breast cancer [150].

Emerging technologies, such as artificial intelligence (***AI***) and machine learning, further enhance this concept. AI‐based systems integrate genomic, epigenomic, and transcriptomic data to identify predictive autophagy‐epigenetic modifications and optimize drug combinations [151]. Tools such as **i‐Biomarker CaDx** utilize circulating ***miRNA*** profiles to detect breast cancer accurately and provide therapy guidance [152]. Additionally, AI‐enhanced imaging biomarkers can predict tumor risk and aggressiveness from mammography or pathology slides. As these technologies continue to develop, they hold the potential to speed up therapy optimization and facilitate truly personalized treatment strategies in breast cancer [153].

## 6 Challenges and Future Perspectives

The clinical application of epigenetics and autophagy in breast cancer remains a complex area. While promising mechanistic insights are emerging, translation into clinical practice is slowed down by biological, technical, and therapeutic limitations [154].

**FIGURE 4:**
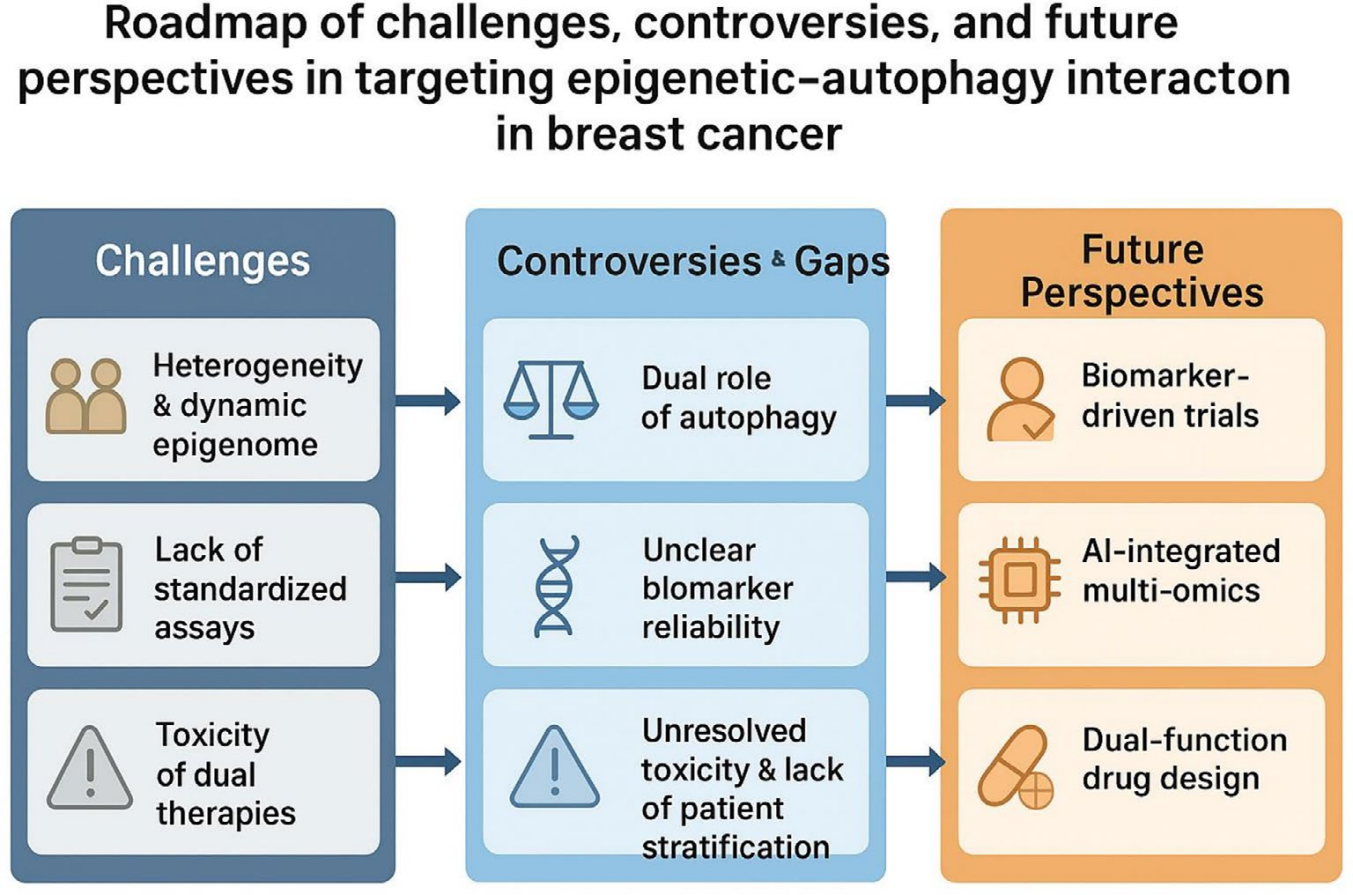
Roadmap of challenges, controversies, and future perspectives in targeting epigenetic–autophagy interactions in breast cancer.

Key limitations include tumor heterogeneity, lack of standardized biomarker assays, and unresolved questions about the dual role of autophagy across different stages of breast cancer. At the same time, rapid technological advances (e.g., **nanopore sequencing** and **scATAC‐seq**) and integrative approaches hold potential to overcome these barriers and refine precision oncology strategies [155]. A roadmap of challenges, controversies, and future perspectives is provided in Figure 4.

### 6.1 Need for Better Biomarker Validation in Clinical Settings

Although numerous pre‐clinical studies have identified DNA methylation signatures, histone modifications, and ***non‐coding RNAs (ncRNAs)*** regulating autophagy, the clinical validation of these biomarkers remains limited. Current clinical trials rarely integrate autophagy‐epigenetic markers into patient selection or therapeutic monitoring, which limits personalized treatment strategies [156].

Key challenges include the absence of standardized, reproducible assays for detecting autophagy‐related epigenetic changes in clinical samples and the impact of tumor heterogeneity, which limits extrapolation across breast cancer subtypes. Moreover, dynamic shifts in the tumor epigenome and autophagy flux during therapy highlight the need for longitudinal sampling and adaptive biomarker panels [157].

Future clinical trials must prioritize validated biomarker‐driven stratification strategies, incorporating methylation panels, ***miRNA*** profiles, and histone modification signatures to guide therapeutic decisions and optimize therapeutic outcomes [47].

### 6.2 Technological Advancements in Epigenomic Profiling for Precision Medicine

Technological innovations are transforming the ability to profile autophagy–epigenetic interactions at high resolution [158]. Approaches such as ***ATAC***‐***seq*** for chromatin accessibility, ***ChIP‐seq*** for histone modifications mapping, and bisulfite sequencing for DNA methylation have revealed critical regulatory networks that underlie breast cancer progression and therapy resistance [159].

Single‐cell epigenomics (like ***scRNAseq***, ***scATAC‐seq***, and ***scNMT‐seq***) represents a powerful advance, enabling the dissection of intratumoral heterogeneity and the identification of rare subclones with distinct autophagy‐epigenetic signatures [160]. In parallel, liquid biopsy approaches facilitate non‐invasive detection of circulating tumor DNA (***ctDNA***) and autophagy‐associated ***ncRNAs***, supporting real‐time monitoring of therapeutic response [161].

Integration of these datasets with AI and machine learning holds promise for predictive biomarker discovery, target identification, and refinement of patient‐specific therapeutic algorithms in breast cancer [162].

### 6.3 Controversies and Knowledge Gap

Despite advancements, several unresolved issues persist. One key controversy arises about the dual role of autophagy: although its suppression via DNA methylation or histone alterations can lead to the onset of tumors, its reactivation in later stages of the disease aids in survival amid chemotherapy‐related stress. This context‐dependent nature makes it difficult to determine the appropriate therapeutic approach regarding whether to stimulate or suppress autophagy at different stages of breast cancer [120, 163].

Another discussion concerns the clinical reliability of epigenetic and ***ncRNA*** biomarkers. While biomarkers like ***BECN1, LC3B, and ATG5*** methylation, along with ***ncRNAs*** such as ***HOTAIR*** and ***miR‐34a***, show potential, their ability to predict outcomes varies across different cohorts and subtypes, particularly in ***TNBC***. It is still uncertain whether these molecules are more effective as therapeutic targets or as indicators for diagnosis and prognosis [164].

Therapeutically, epigenetic modulators combined with autophagy inhibitors have shown strong preclinical synergy, but their translation into the clinic is limited by toxicity, variable efficacy, and the absence of reliable stratification methods [165]. Furthermore, computational and AI‐driven predictions of novel targets require robust biological validation before clinical adoption [166].

### 6.4 Novel Drug Discovery Efforts Combining Autophagy and Epigenetic Modulators

Therapeutic strategies targeting both autophagy and epigenetic regulation are entering a dynamic phase of drug discovery. Preclinical studies strongly support the synergistic anti‐tumor effects of combining epigenetic drugs, such as ***DNMT*** inhibitors or ***HDAC*** inhibitors, with autophagy inhibitors like **Chloroquine** or **Hydroxychloroquine**, particularly in therapy‐resistant triple‐negative breast cancer [167]. However, optimizing dosing regimens and sequencing remains complex, and the stage‐dependent role of autophagy adds another layer of difficulty.

Future drug discovery efforts are increasingly focused on next‐generation small‐molecule targeting key autophagy effectors (e.g., ***VPS34***, ***ULK1*** inhibitors) alongside novel epigenetic modulators with improved specificity and reduced toxicity. An emerging frontier involves the development of dual‐function compounds capable of simultaneously modulating chromatin accessibility and autophagic flux, offering the potential for precision‐tailored therapeutic regimens [168].

## 7 Conclusion

Based on the literature available, this review highlighted the complex interplay between autophagy and epigenetic regulation in breast cancer, stressing their combined roles in therapy resistance, tumor progression, and therapeutic/clinical outcome prediction. Epigenetic alterations such as DNA methylation, histone tail modifications, and ncRNAs modulate genes related to autophagy, thoroughly affecting tumor cell survival under treatment stress. Aberrant regulation of epigenetic and autophagic pathways not only contributes to tumor aggressiveness but also shows a diverse connection between the development of novel treatment strategies and cancer biomarkers.

Innovations in epigenomic profiling and liquid biopsy technologies, along with the identification of actionable epigenetic signatures related to autophagy, are creating opportunities for personalized medicine strategies in breast cancer. Additionally, the development of combination therapies that focus on both epigenetic regulators and autophagy pathways shows great promise in addressing therapy resistance, especially in aggressive forms like ***TNBC***. Future initiatives that incorporate biomarker‐driven patient classification, technological advancements, and AI‐enhanced therapy optimization will be essential for converting these findings into lasting clinical advantages. Collectively, a more profound comprehension of the interaction between epigenetics and autophagy has the potential to transform breast cancer treatment and enhance patient outcomes.

## Author Contributions

The author, Bushra Faryal wrote and edited the manuscript. Data authentication is not applicable. The author has read and approved the final manuscript.

## Ethics Statement

The author has nothing to report.

## Consent

The author has nothing to report.

## Conflicts of Interest

The author declares no conflicts of interest.

## Data Availability

Data sharing not applicable to this article as no datasets were generated or analysed during the current study.
